# Shaping of Shared Autonomous Solutions With Minimal Interaction

**DOI:** 10.3389/fnbot.2018.00054

**Published:** 2018-09-03

**Authors:** Christopher Reardon, Hao Zhang, Jonathan Fink

**Affiliations:** ^1^United States Army Research Laboratory, Adelphi, MD, United States; ^2^Department of Computer Science, Colorado School of Mines, Golden, CO, United States

**Keywords:** shared autonomy, artificial intelligence, robotics, human-robot interaction, autonomous surveillance

## Abstract

A fundamental problem in creating successful shared autonomy systems is enabling efficient specification of the problem for which an autonomous system can generate a solution. We present a general paradigm, Interactive Shared Solution Shaping (IS3), broadly applied to shared autonomous systems where a human can use their domain knowledge to interactively provide feedback during the autonomous planning process. We hypothesize that this interaction process can be optimized so that with minimal interaction, near-optimal solutions can be achieved. We examine this hypothesis in the space of resource-constrained mobile search and surveillance and show that without directly instructing a robot or complete communication of a believed target distribution, the human teammate is able to successfully shape the generation of an autonomous search route. This ability is demonstrated in three experiments that show (1) the IS3 approach can improve performance in that routes generated from interactions in general reduce the variance of the target detection performance, and increase overall target detection; (2) the entire IS3 autonomous route generation system's performance, including cost of interaction along with movement cost, experiences a tradeoff between performance vs. numbers of interactions that can be optimized; (3) the IS3 autonomous route generation system is able to perform within constraints by generating tours that stay under budget when executed by a real robot in a realistic field environment.

## 1. Introduction

To create a shared autonomy system, a critical challenge is to correctly and succinctly specify the problem space from which a solution can be autonomously generated. Much of the problem-specification challenge arises from the central issue of translating a human's mental model of the problem into a format that can be reasoned upon by the autonomous robot. We believe that a general paradigm can be broadly applied to shared autonomous systems where a human can use his or her domain knowledge to interactively provide feedback during the autonomous planning process, i.e., providing hints or suggestions about the underlying model of the system. We refer to this paradigm as Interactive Shared Solution Shaping (IS3).

Importantly, we believe this interaction process can be optimized so that with minimal interaction, near-optimal solutions can be achieved. We examine this hypothesis in the space of resource-constrained mobile search. Specifically, we address the problem of construction of path or tour of the environment so that a robot equipped with a visibility-based sensor will optimally detect targets that appear in the environment, subject to the challenge of limited-resource constraints (e.g., time, energy) imposed by real-world applications.

This resource-constrained search task is closely related to the class of informative path planning problems (Kollar and Roy, 2008; Julian et al., 2013), where path planning for exploration is achieved via maximization of mutual information gain. Information-theoretic methods were also used to provide a mathematical basis for autonomously optimizing target-detection trajectories (Charrow et al., 2015b). This task is also deeply connected to the class of problems referred to as the selective traveling salesperson problem or orienteering problem (Laporte and Martello, 1990). Recent work proposed the correlated orienteering problem, where the rewards for visiting locations are not independent, along with candidate solution algorithms that are well-suited to robotic information gathering (Yu et al., 2014; Arora and Scherer, 2016). However, these methods assume (1) a fully-specified problem definition, and (2) no human teammates are in the planning loop.

The IS3 paradigm, in which the robot's decision making is shaped by the human's knowledge through minimal interaction, is a form of shared autonomy in that planning and decision making are shared by the human and the autonomous agent, using the strengths of each to arrive at a better solution. For this work, we ground this idea experimentally in the form of path planning for mobile search, where the route planning is shared to achieve better performing search routes. The planned routes are executed to evaluate the performance after interaction.

The primary contributions of this work are the general introduction of our IS3 paradigm and the specific application to the autonomous route planning problem to show that with minimal interactions, a human teammate's partial knowledge can be shared to improve autonomous route planning. This is demonstrated in three application scenarios:

1. We show in a resource-constrained surveillance tour generation scenario how the IS3 approach can improve performance in tours generated from interactions reduce the variance of the target detection performance, and increase overall target detection.
2. To examine the cost effectiveness of interaction, we treat each interaction and corresponding re-planning as part of the cost of the end-to-end system performance, and examine optimal performance with numbers of interactions in the context of informative path generation.
3. We implement an IS3 autonomous route planning system on a real robot in a realistic field environment, and show the system is able to perform within constraints by generating tours that stay under budget when executed.

The remainder of this paper is organized as follows. Section 2 covers related work in shared autonomy for autonomous planning and information gathering; section 3 details the general context, problem statement, and approach; section 4 delves into a specific formulation, approach, and experiments on how IS3 can be used for improving performance in resource-constrained generation of surveillance tours; section 5 expands the problem formulation and approach as well as experiments that show the effect of incorporating interaction cost as part of the end-to-end system performance; section 6 demonstrates the IS3 approaches application to performance within constraints by generating tours that stay under budget when executed by a real robot in a realistic field environment; section 7 concludes the work.

## 2. Related work

At its core, this work is closely related to the general problem of planning informative paths for mobile robots. Most commonly, the application of interest is map-exploration, i.e., autonomous uncovering of environment structure by planning trajectories that maximize information gain on the underly probabilistic map representation. Recent methods form this as optimization-based solutions to problems of active control and planning as presented by Kollar and Roy (2008), Julian et al. (2013) and Charrow et al. (2015a). More recently, similar information-theoretic techniques have been applied to the target-detection and tracking problems highlighted by Dames et al. (2015) and Charrow et al. (2015b).

The above techniques are all considered in the paradigm of receding horizon control. That is, they operate in the context of a feedback controller, reacting to the most recent model of the environment or problem at hand. When the goal is to autonomously plan for the best sequence of actions over a longer, possibly infinite, time horizon, it is common to turn to techniques from the Operations Research (OR) community. This is especially true when one seeks to incorporate budget- or topologically-based constraints. Further, in these settings, it is typical to have a discrete rather than continuous representation of locations of interest in the environment. For example, the traveling salesperson problem described by Laporte and Martello (1990) looks to find the shortest path that visits all sites, forming a *tour* that returns to the starting location.

When a budget is introduced to this problem it is referred to as the selective traveling salesperson or Orienteering Problem (OP). It is well known that this is an NP-hard problem and most algorithms addressing the OP rely on approximations. Indeed, the development of practical solution algorithms continues to be an active area of research (e.g., Blum et al., 2007; Vansteenwegen et al., 2011). While solutions from the OR community typically focus on problems with fairly coarse discretizations of the environment, recent work by Tokekar et al. (2016) has demonstrated how these techniques can be applied in the field-robotics domain for hybrid aerial-ground systems.

In addition to the assumption of pre-computed discrete sites, traditional solutions to the OP problem typically also assume independent reward at each site. However, in a real-world information-collecting application, it is clear that rewards for visiting sites, especially nearby ones, are highly correlated. Indeed, this observation was noted by Yu et al. (2014) where the *correlated* orienteering problem is introduced as an extension where the reward for visiting each location is correlated with the set of other locations visited, making the problem more amenable to planning informative tours in the context of persistent monitoring. More recently, Arora and Scherer (2016) demonstrate efficient approximate algorithms that solve this problem at speeds making it reasonable to use in an online robotic setting. We adopt the structure of this algorithm in our work here.

One of the key observations of this work is that in all of the above planning and control scenarios, the robot or autonomous planning system has a precise definition of the objective function. There has been considerably less attention paid to how a human operator or teammate can efficiently communicate this objective function to the autonomous system.

There is, however, some work by Crossman et al. (2012), Alonso-Mora et al. (2015), and Dawson et al. (2015) that does looks toward human interaction with autonomous planning systems. We see two fundamental and contrasting approaches that are taken. First, work such as that of Yi et al. (2014) models human input as a sequence of constraints within which the system plans for an maximally informative path. Second, in the work by Lin and Goodrich (2010) a strategy is adopted where the human shapes the objective function that is used to make autonomous decisions. Since we are interested in a domain of problems that are already heavily constrained, e.g., with limited budget and requirements on cyclical paths, we adopt the second strategy and focus on how the human teammate can provide *iterative* updates to the objective function, demonstrated as a proof-of-concept in our earlier work Reardon and Fink (2017).

Finally, we do note that there is a potential connection between our work and the work concentrating on the idea of *reward shaping* in the reinforcement learning community. Clearly, there is a fundamental difference in the objective when interacting with a system during the *training* of a policy rather than the *execution* of an autonomous planning algorithm. However, we do draw inspiration from the interactive approaches described by Judah et al. (2014) and Raza et al. (2015).

## 3. Problem formulation and approach

We are interested in planning resource-constrained informative routes for a mobile robot with a visibility-based sensor, e.g., a camera or laser range-finder, in complicated environment. For example, we specifically consider the Clearpath Robotic's Jackal platform depicted in Figure 9 in environments such as the one depicted in Figure 10. We assume that the mobile robot has simultaneous localization and mapping (SLAM) capabilities that allow it to autonomously generate and navigate along collision-free trajectories or motion plans in an unstructured environment. The goal of this work is to choose a motion plan for the robot that maximizes the probability of detecting a target, e.g., a victim in the disaster response scenario or threat in the military domain. While the robot may have a coarse prior for target locations, the key idea of this work is that a human teammate can leverage prior knowledge, experience, and sensory cues to infer a higher-fidelity distribution for target locations.

**Figure 1:**
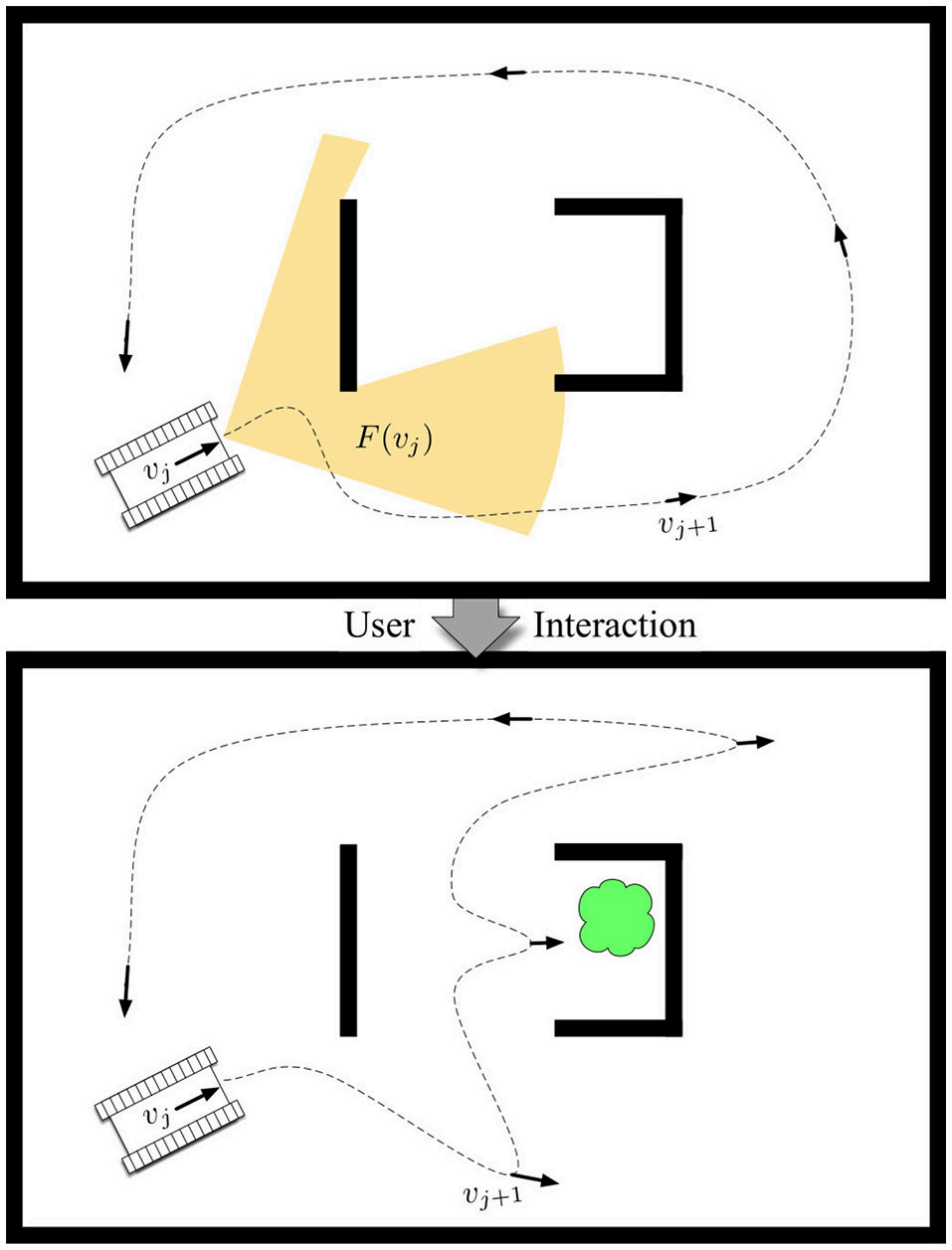
The problem of resource-constrained surveillance is to find a set of viewpoints *v*_*j*_ that maximize the expected target-detection rate based on sensor footprints *F*(*v*_*j*_) such that a path can be driven to visit all viewpoints by a mobile robot within a cost budget *B*. The contribution focuses on the novel formulation and approach of solving this problem in a human-robot teaming scenario, in which a human interacts with the robotic system by adjusting its prior belief on target locations (e.g., the cloud) to achieve information-gathering tours that are high-performing.

Mathematically, we can model the robot's prior on target locations as an occupancy grid **g** consisting of a set of *G* independent cells {*g*_1_, …, *g*_*G*_} such that the probability of there being a target in cell *i* is *p*(*g*_*i*_ = 1). Then, if the robot achieves a viewpoint in the environment, *v*_*j*_ ∈ *SE*(2), i.e., *v*_*j*_ = [*x, y*, *θ*], the visibility-based sensor will observe a set of cells *g*_*i*_ ∈ *F*(*v*_*j*_) as depicted in Figure 1. We note that *F*(*v*_*j*_) can be generally computed based on properties of the sensor, e.g., field of view and maximum range, along with a map of the physical environment. We also assume a common heuristic from information-theoretic exploration ( Charrow et al., 2015b), that observations are taken at each viewpoint, and adjust the granularity of our candidate viewpoints to one that is appropriate to the size of the robot's sensor footprint to ensure the heuristic is accurate for path performance.

Let $q_{i}^{j}$ be the measurement made of cell *g*_*i*_ from viewpoint *v*_*j*_, then the target detection model from Charrow et al. (2015b) can be denoted as: 

(1) $$\begin{array}{l} p(q_{i}^{j}=1|g_{i}=1)=\gamma \;p(q_{i}^{j}=0|g_{i}=1)=1-\gamma \\ p(q_{i}^{j}=1|g_{i}=0)=0\;p(q_{i}^{j}=0|g_{i}=0)=1. \end{array}$$

Note that this model assumes no false-positive measurements and a true-positive rate of γ.

Since our goal is to maximize the probability of obtaining positive observations of targets in the environment, we define the *reward* for a viewpoint *R*(*v*_*j*_) to be the expected number of target detections,

(2) $$R(v_{j})=\sum_{g_{i}\in F(v_{j})}p(g_{i})\cdot p(q_{i}^{j}|g_{i}).$$

While the probability of a target in each cell is independent, the probability of target presence for a set of measurements given the occupancy grid, e.g., *p*(**q**|**g**), is not independent, as observations overlap. However, for a binary sensor with high true-positive rate *γ*, we can closely approximate by only considering the first observation of each cell *g*_*i*_. This means that for a set of observations **v** = {**v**_*j*_}, we can write the reward *R*(**v**) as

(3) $$R(\text{v})=\sum_{g_{i}\in G_{\text{v}}}p(g_{i})\cdot p(q_{i}^{j}|g_{i})$$

where *G*_**v**_ = {*F*(*v*_1_) ∪ *F*(*v*_2_) ⋯ ∪ *F*(*v*_*j*_)}.

Then, the resource-constrained informative path planning problem can be defined as an optimization problem to find a sequence of viewpoints [**v** = *v*_1_, …, *v*_*N*_] subject to application-specific constraints. In particular, we consider a class of problems that constrain the total duration of the planned path. That is, given a cost of traversal between two viewpoints as *C*(*v*_*i*_, *v*_*i*+1_) > 0 and total path cost $C\left(\text{v}\right)=\underset{i\in 1,\ldots ,N,1}{\sum }C\left(v_{i},v_{i+1}\right)$, we introduce a constraint *C*(**v**) ≤ *B* and write a general optimization problem as

(4) $$\begin{array}{l} \underset{\text{v}\subset V}{\arg\max}\;R(\text{v})\\ \text{subject to }C(\text{v})\leq B. \end{array}$$

This problem is the basis for the remainder of this work.

It is important to note two characteristics of Equation (4) that make it challenging to compute. First, from Equation (3), it is clear that the sum of independent rewards *R*(*v*_*j*_) is an upper bound for the actual reward *R*(**v**), i.e., $\underset{v_{j}\in \text{v}}{\sum }R\left(v_{j}\right)\geq R\left(\text{v}\right)$. This means the value of a route must be computed in aggregate. We employ several of the techniques from Charrow et al. (2015b) to improve runtime, e.g., caching of ray-trace computations. Second, the selection of viewpoints **v** is inherently a continuous search problem over the space of all robot poses and includes the planning of trajectories between viewpoints to compute the route cost *C*(**v**). We address this by approximating the space of routes with the construction of a probabilistic road map (PRM) technique, abstracting the continuous route planning problem to a lower-dimensional graph search.

Solving Equation (4) is not inherently novel. Indeed, solutions to similar problems are the core of wide swaths of the robotics and operations research literature. However, recall the context in which we are operating: the robot only has a coarse, most likely inaccurate or naive, model of likely target locations that directly influence the values of *R*(**v**) while a human teammate can perform higher-order inference to estimate more accurate target priors. This high-level human inference could be imagined as resulting in a continuous distribution over a complex environment, synthesized from an array of information sources (e.g., history, experience, and observations). Given its complexity, the complete communication of this prior is likely intractable.

**Figure 2:**
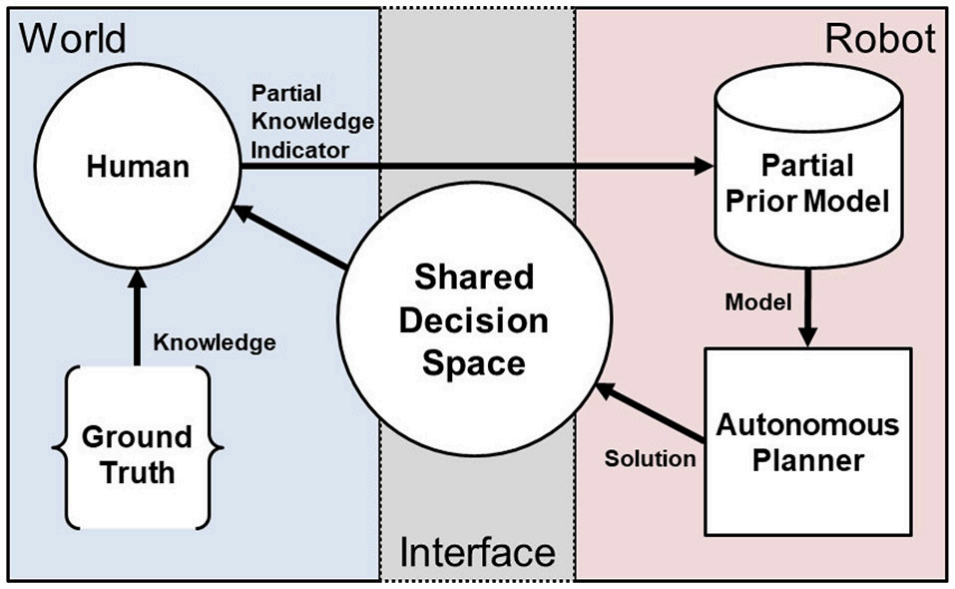
An overview of the IS3 approach. The human uses knowledge of the ground truth of the world domain state, e.g., in the form of objectives, prior experience, observations, etc., to provide partial indicators of that knowledge to the robot through minimal interaction and arrive at a shared problem space definition that is specified sufficiently to maximize performance.

Our general approach is to use interactions where the human teammate iteratively provides partial indicators of the ground truth of the shared decision space to the autonomous system as depicted in Figure 2. Using these partial indicators, the system regenerates an autonomous solution to the route planning problem in Equation (4). In this way, the human teammate is able to shape the generation of autonomous solutions in the shared decision space without directly instructing the robot and without complete communication of the ground truth. We employ this interactive approach in three illustrative scenarios to examine:

1. How does route shaping affect performance? (Section 4)
2. What is the effect of incorporating interaction and replanning as part of the overall budget? (Section 5)
3. When deployed on a real-world robot surveillance application, can the IS3 system plan and execute routes while remaining under budget? (Section 6).

## 4. Performance in resource-constrained tour generation

First, we show that the IS3 approach can improve performance. We examine the problem of continuous surveillance with the added constraint that the surveillance route must return to the starting position within the budget *B*, i.e., a “survillance tour.” To address the autonomous surveillance tour problem, we introduced Human-Autonomous Route Planning (HARP) in Reardon and Fink (2017), which explores the space of surveillance solutions to maximize task performance. The optimal surveillance route generation algorithm HARP uses is a modification of the algorithm for Random Orienteering inspired by (Arora and Scherer, 2016) shown in Algorithm 2. Our results show a performance increase for human-robot interaction vs. a baseline method with no interaction by measuring the target detection rate over a large sample set for a complex simulated environment.

### 4.1. Approach

To forumate the surveillance route as a tour, we modify Equation 4 to include the constraint that **v** must end with *v*_*s*_, the starting point:

(5) $$\begin{array}{l} \underset{{}_{\text{v}\subset V}}{\arg\max}\;R(\text{v})\\ \text{subject to }C(\text{v})\leq B.\\ \text{ v}=\left[\begin{array}{c} v_{s},\ldots ,v_{s} \end{array}\right]. \end{array}$$

Introducing this constraint casts the problem as a correlated orienteering problem (Yu et al., 2014).

Our algorithm begins with first, given an uninformed (e.g., uniform) target belief prior **g**^0^, a set of candidate viewpoints is selected, and a solution to the correlated orienteering problem Equation (4) is constructed. This initial solution **v**^0^ is presented graphically as an under-budget surveillance tour of the environment. Then, the human is able to modify the target belief prior with a single interaction to create **g**^1^. This new **g**^1^ represents a better-informed prior, and a solution to the correlated orienteering problem is generated using this new information and **v**^0^. The human teammate can then view and provide successive interactions to shape the surveillance route generated.

**Figure 3:**
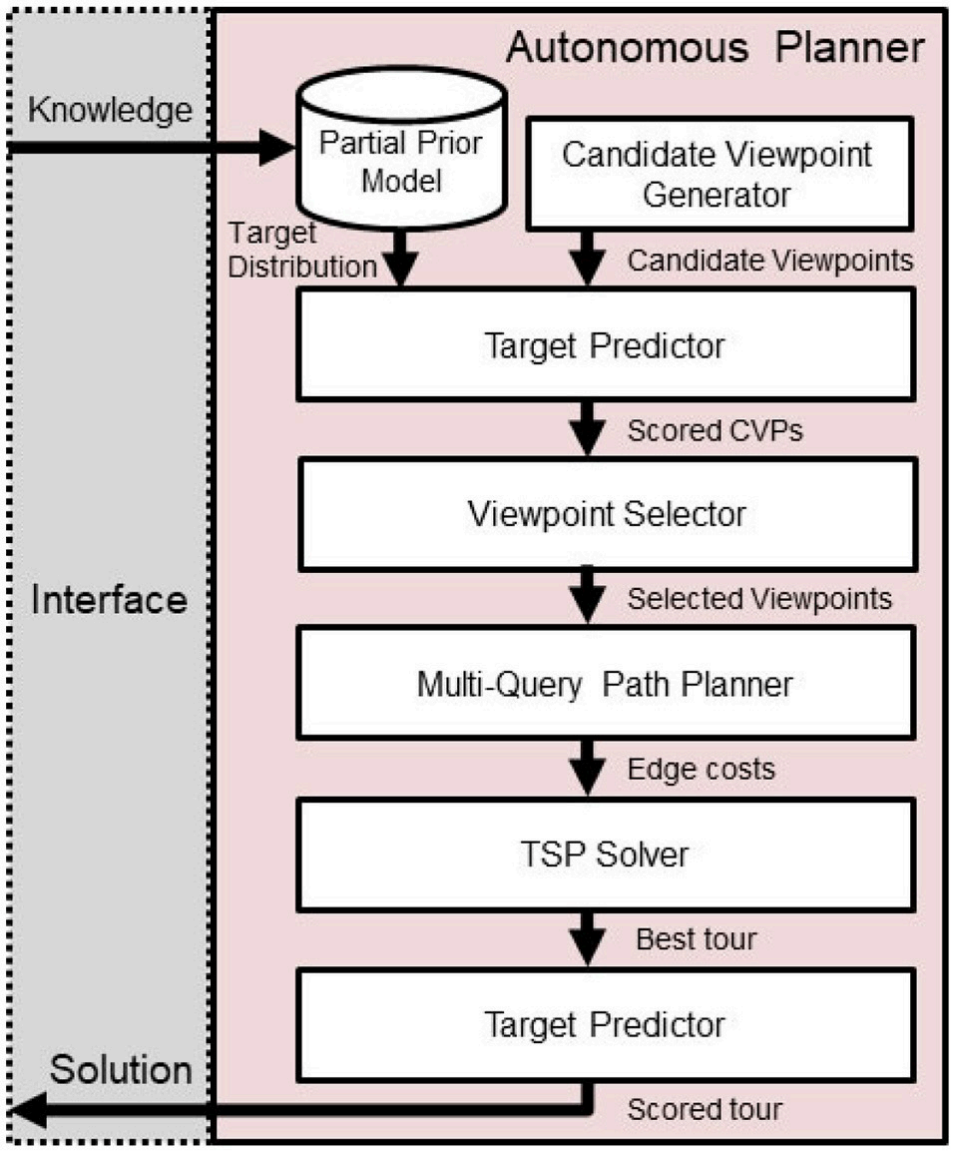
Route planner overview. Progress flows downward, beginning with human input and generated solutions will be presented back to the human teammate.

As graphically illustrated in Figure 3, a set of candidate viewpoints *V* = {*v*_*j*_ =[*x, y*, *θ*]}are generated by sampling over unoccupied space within the environment. Then, each candidate viewpoint's reward *R*(*v*_*j*_) is scored individually based upon the target belief as defined in Equation (2). Because the actual detection rates of novel targets are dependent on observations from other viewpoints, these scores serve as an upper bound on the reward for visiting each viewpoint *v*_*j*_.

While the correlated information-gain based reward function is sub-modular and therefore efficient optimization solutions can be devised, with the addition of a traveling budget constraint, this problem becomes non-submodular (Arora and Scherer, 2016). To address this issue, after the *Prepare Input* step is complete, we split the correlated orienteering problem into a combination of Constraint Satisfaction and Traveling Salesperson problems. Then, we implement a new approach by adapting and modifying the Random Orienteering (RO) algorithm Arora and Scherer (2016), as shown in Algorithm 2.

In the new Algorithm 2, we iteratively explore subsets of candidate viewpoints **v** ⊂ *V*, i.e., the Constraint Satisfaction Problem, and then checking for a tour within the cost budget *B*, which is the Traveling Salesperson Problem (TSP).

We note that the construction of the edge weights for a TSP in a realistic robotics application can be computationally expensive in its own right and involves motion planning with respect to complicated environments and differential constraints. Thus, we address this problem in three ways: (1) evaluating edge costs for only the subset of viewpoints being considered, (2) caching path queries, and (3) leveraging algorithms shown successful in the “multi-query” setting, e.g., the probabilistic roadmap method Kavraki et al. (1996). In this way, we spend some precomputation effort to speed up later calculations of the cost to traverse from one viewpoint to another, *C*(*v*_*i*_, *v*_*j*_). We assume the costs between viewpoints to be symmetric such that *C*(*v*_*i*_, *v*_*j*_) = *C*(*v*_*j*_, *v*_*i*_).

One challenge of implementing and applying Algorithm 2 is that the rate of convergence to a solution is influenced by the initial chosen set of viewpoints. We overcome this by introducing an initialization method, shown in Algorithm 1, which performs sampling of *m* candidate viewpoints, weighted by reward, to initially explore several disparate solutions with high reward upper bounds and continuing with the one that is under budget and maximizes reward. We score the actual reward *R*(**v**) for a candidate viewpoint selection based upon Equation (3), which accounts for coverage overlapping between viewpoints and provides an accurate representation of the target detection rate.

**Algorithm 1:**
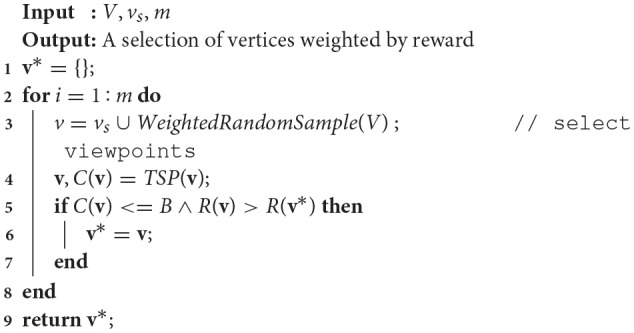
Sample weighted random solutions (Reardon and Fink, 2017).

Algorithm 2 continues by randomly sampling candidate viewpoints *v*_*j*_ ∈ *V* (line 9), updating the active solution **v** (lines 10–15), and evaluating with respect to the current-best solution tour v^*^ (line 16). If the viewpoint selected is currently in the tour **v**, it is removed (line 11); if it is not in the tour, then it is added (line 13). If the cost of **v** is under budget and it improves the reward over v^*^, it is kept as the current best tour (line 17).

**Algorithm 2:**
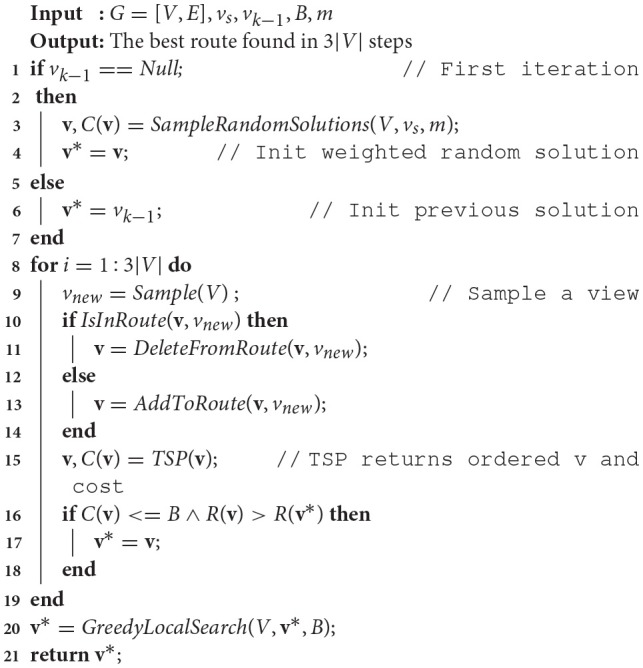
Modified RO based on Arora and Scherer (2016) for solving decoupled constraint satisfaction and TSPs from previous work (Reardon and Fink, 2017).

After 3|*V*| iterations (per the standard probabilistic constraint satisfaction problem algorithm from Schoning, 1999 modified in Arora and Scherer, 2016), a modified version of the Greedy Local Search from Arora and Scherer (2016) is performed (Algorithm 3, where δ is a distance threshold and *c* is a reward threshold) to improve anytime performance by incorporating nodes in the neighborhood of the chosen route that increase the reward over a threshold value, *c*, while remaining under cost. First, a list of eligible candidates within a distance threshold δ of existing tour viewpoints is constructed (lines 1–6). Then, if the addition of any of those candidates increases the tour reward *R*(v^*^) while being under budget *B* (lines 7–11), the viewpoint is added to the tour. Finally, after the greedy local search is complete, the v^*^ tour is returned.

**Algorithm 3:**
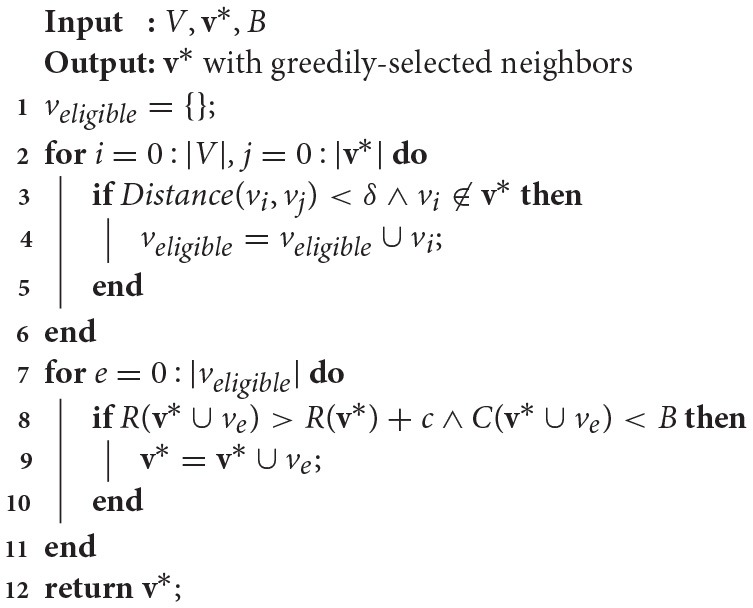
Modified greedy local search (Reardon and Fink, 2017).

### 4.2. Experiments

First, we show that the IS3 approach can improve performance. We examine the performance increase for human-robot interaction vs. a baseline method with no interaction by measuring the target detection rate over a large sample set for a complex simulated environment.

#### 4.2.1. Setup

A complete end-to-end surveillance route generation system has been implemented as a suite of software modules leveraging ROS. To visualize the environment, as well as the current target belief prior, tour solution, and regions observable by the robot, we leverage the RViz tool. The user is able to edit the target belief prior using custom plugins by “painting” regions of higher target probability with the mouse pointer. After such interaction, the system uses an implementation of our approach to regenerate a new surveillance tour solution using the new prior given in the interaction.

Rather than conduct a large-scale user study, we simulate human input: given a hidden underlying ground-truth target distribution, our human-simulator generates a set of interactions ordered based on the greatest size and probability value of each area of elevated probability in the ground truth, then communicates each interaction via the same mechanism as the human interface.

**Figure 4:**
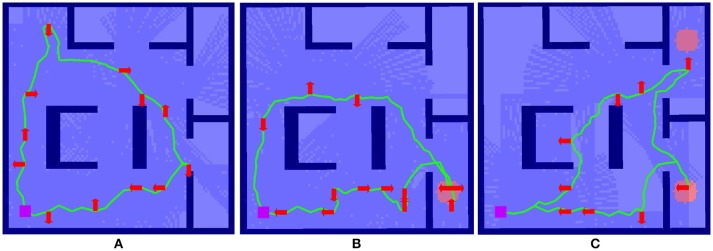
Example of the effect of interaction on route surveillance generation in a complex environment visualized in RViz. **(A)** shows the baseline case, where a route is generated autonomously with a uniform target distribution. **(B)** shows the impact of a single interaction, where a route after the target distribution prior is modified to elevate an area of higher target probability, shown as the red circle. **(C)** shows the autonomous route generated after two interactions.

For this experiment we use an orthogonal environment, as illustrated in Figure 4. For these experiments, budget values are selected empirically to create a realistic and challenging scenario, i.e., we constrain the resources available (i.e., the budget, *B*) so that full coverage of the map is not possible.

#### 4.2.2. Results

Here we summarize the results originally presented in Reardon and Fink (2017). We illustrate the effect of solution shaping on performance in Figure 4. The case of no interaction in Figure 4A serves as a baseline; a circular route generated from a uniform prior target distribution **g**_0_ achieves a reasonable tour of the environment, subject to the budget *B*.

In Figure 4B, the tour generated from a single interaction **g**_1_ where a partial indicator of higher probability of target presence has been specified in the lower righthand room. A new route is generated using Algorithm 2 and the route shown in Figure 4A as a starting point. This new route reprioritizes fully cover the lower right room over upper left, an outcome that was made without explicit instruction by the human.

Figure 4C shows an even further shaping effect, as a second partial indicator shows a higher target probability in the upper right for **g**_2_. This results in a replanning that focuses entirely on the two areas of higher probability. In this way, the human is able to use minimal interactions shape the decision space for autonomous route generation without explicit instruction.

**Figure 5:**
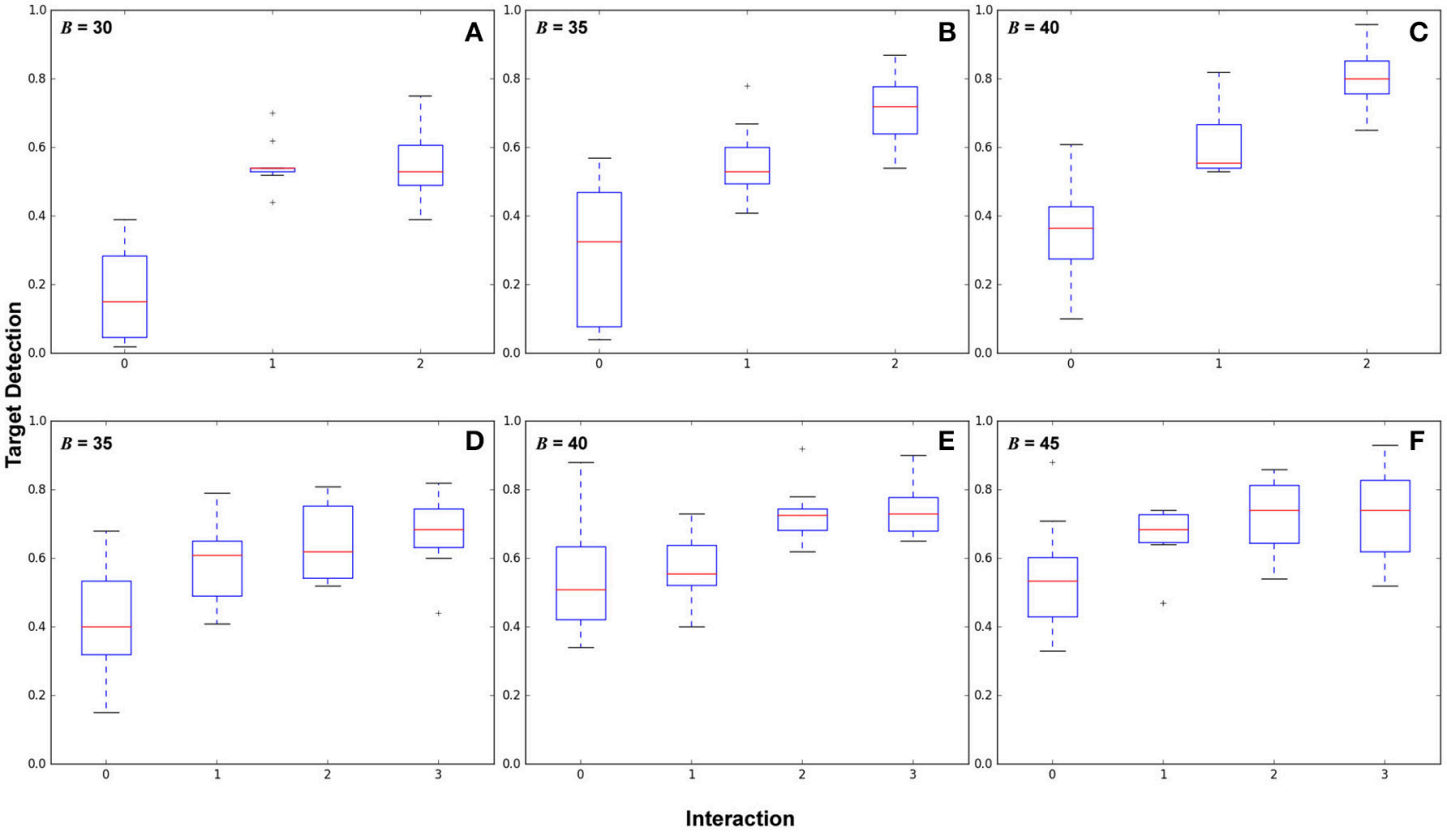
Results comparing different numbers of human interactions across budget parameters showing target detection performance. The top and bottom of the boxes represent the interquartile range (middle 50% of samples), the red line is the mean, and the whiskers represent the overall value range. **(A–C)** show two areas of high target likelihood with budget *B* = (30, 35, 40), with variance values for 0, 1, and 2 interactions in **(A)**:(0.019, 0.005, 0.011), **(B)**:(0.043, 0.011, 0.012), and **(C)**:(0.022, 0.011, 0.009). **(D–F)** show three areas of high target likelihood with budget *B* = (35, 40, 45), with variance values for 0, 1, 2, and 3 interactions in **(D)**:(0.024, 0.013, 0.011, 0.012), **(E)**:(0.023, 0.010, 0.006, 0.007), and **(F)**:(0.024, 0.009, 0.011, 0.017).

Figure 5 shows box plots of 210 experiments, 10 per plot. Each box represents a number of interactions. Figures 5A–C shows from baseline (no interactions) to *n* = 2 interactions with budget values *B* = 30, 35, 40 and Figures 5D–F shows 0–3 interactions with budget values *B* = 35, 40, 45.

These results show that routes generated from interactions in general reduce the variance of the target detection performance, and increase overall target detection. Significant differences in performance (*p* < 0.05) were found between each interaction. Variance decreased over baseline for all cases. We also make three observations: (1) In some cases there is a point of diminishing returns with respect to interactions, for example in Figure 5A where *B* = 30, between interactions 1 and 2, in Figure 5E between interactions 2–3, and in Figure 5F between 1 and 3. (2) Observing this diminishing returns effect, in cases where autonomous surveillance routes will provide sufficient coverage from only a partial belief prior, it may not be necessary to provide an exhaustive target belief prior to the robot. (3) the point at which returns diminish is subject to the task definition and the experimental configuration, including budget, environment, target likelihood, and sensor footprint.

## 5. Optimizing interaction as part of cost

For the IS3 approach to be practical for use in real-world robotics, the interaction component cannot be a slow process. In the context of resource-constrained search, the cost of interaction cannot overwhelm the budget. Therefore, we extend (Reardon et al., 2017) by examining the compelling situation where a human-robot team must locate a number of targets in the environment prior to a potentially catastrophic event. This event could be, e.g., before a disaster victim expires, before environmental events hinder search, or even something as extreme as locating a timed explosive device before detonation. We refer to these collectively as “ticking time-bomb” scenarios, where the resource limitation is imposed not only on the robot (e.g., the battery life of a ground robot or small UAV) but on the entire IS3 autonomous route planning system's start-to-finish operation. The challenge then is to not only generate an under-budget path as in Reardon et al. (2017), but also to incorporate the cost of solution shaping.

This interdependent combination of planning and execution has not been previously addressed and is highly relevant to real-world robotics problems. We introduce a novel formulation and approach to address this combination.

### 5.1. Approach

To formulate interaction cost as part of total cost, we introduce a modification of Equation (4) that incorporates the cost of each interaction *k*_*i*_, where *K* is the sum of the cost of all interactions, $K=\underset{i\in 1,\ldots ,n}{\sum }k_{i}$, and rewrite Equation (4) as:

(6) $$\begin{array}{l} \underset{\text{v}\subset V}{\arg\max}\;R(\text{v})\\ \text{subject to }C(\text{v})+K\leq B\\ \text{ v}=\left[\begin{array}{c} v_{s},\ldots \end{array}\right]. \end{array}$$

Equation (6) allows us to balance the cost of interaction and subsequent replanning to achieve a more realistic value of the reward gained when acquiring new information through interaction. In our application we iteratively solve Equation (6) with each interaction using an increasing value for *K*, which is the increasing cost of interaction.

We introduce a modification of the approach from section 4.1. To show the generalizability of the IS3 method, we incorporate an alternate path planner algorithm. A greedy recursive search-based path planner with an *n*-ply lookahead is implemented. In the “ticking time-bomb scenario,” we assume that maximum target detection is critical; i.e., this is a search task not a surveillance task. Therefore, we also relax the assumption of a tour and generate non-circular path.

Algorithm 4 contains pseudo-code for the greedy planner, which recursively traverses all nodes in the graph from the last choice down to a maximum depth *D* to construct a list of candidate choices V^*^ that are under budget *B*. Then, the adjacent node that has the best potential reward considering that lookahead depth is chosen. This process is repeated until the cost of the path constructed exhausts the budget.

**Algorithm 4:**
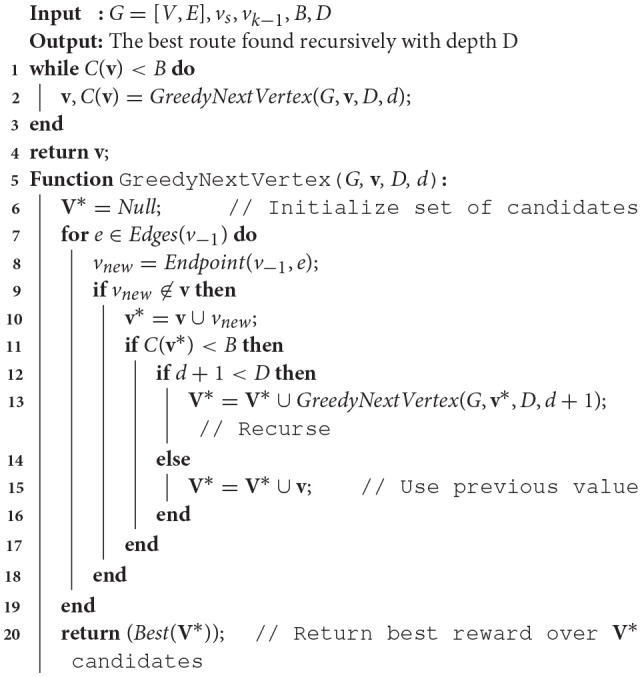
Recursive greedy route planner with lookahead.

### 5.2. Experiments

We conduct experiments to show the performance of our IS3 system in this application subject to the constraints in this new formulation. We examine the utility of interaction in this context to show that there is an optimal number of interactions before performance begins to decline.

#### 5.2.1. Setup

As in section 4, we simulate human input given an underlying ground-truth target distribution which results in a set of interactions based on the size and probability magnitude, which is communicated via the human interface to the planner to generate a new solution. The same end-to-end setup in ROS is used.

**Figure 6:**
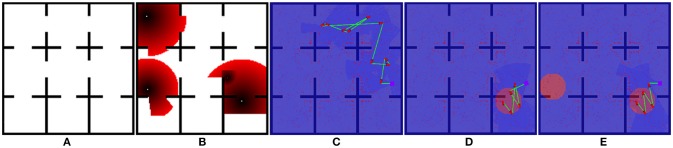
One of the maps used for Optimizing Interaction as Part of Cost experiments (Section 5.2). **(A)** show the original occupancy map. **(B)** shows an example target probability prior superimposed over the occupancy map, with darker red regions indicating higher probability. **(C–E)** show the effect of zero, one, and two interactions in the scenario where interaction is considered as part of the end-to-end cost.

**Figure 7:**
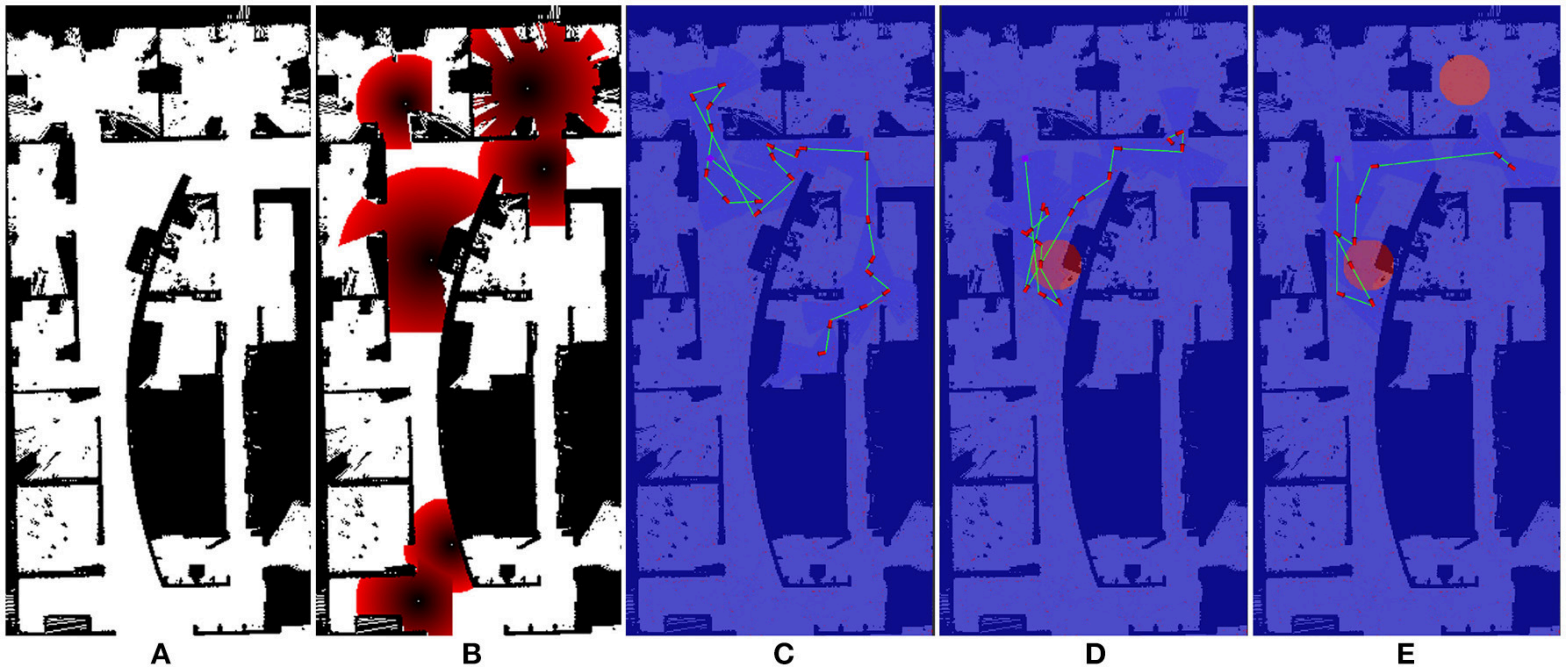
One of the maps used for Optimizing Interaction as Part of Cost experiments (Section 5.2). **(A)** show the original occupancy map. **(B)** shows an example target probability prior superimposed over the occupancy map, with darker red regions indicating higher probability. **(C–E)** show the effect of zero, one, and two interactions in the scenario where interaction is considered as part of the end-to-end cost.

For this experiment, we implement the greedy planner in Algorithm 4 to demonstrate that generalizability of our path shaping approach. We run the experiment using both the orthogonal environment with nine rooms shown in Figure 6A and a more complicated real-world environment shown in Figure 7A. We allow for complex definitions of ground truth by randomly generating new ground truth priors over unoccupied space of varying sizes, which results in a variable number of interactions.

#### 5.2.2. Results

We illustrate the effect of interaction in this scenario in Figures 6C–E, 7C–E. Figures 6B, 7E both show an example target probability prior that has been randomly generated as in the experiments. Similar to Figure 4, the effect of interaction is depicted; however, we employ a greedy planner and generate a non-circular path. Figures 6C, 7C represent the baseline case of no interaction, whereas Figures 6D, 7E of each figure show one and two interactions, respectively. As can be seen, the diminishing budget availability due to the cost of interaction being considered in the end-to-end cost reduces the planner's ability to take advantage of new information, leading to an optimal trade-off point where returns begin to diminish.

**Figure 8:**
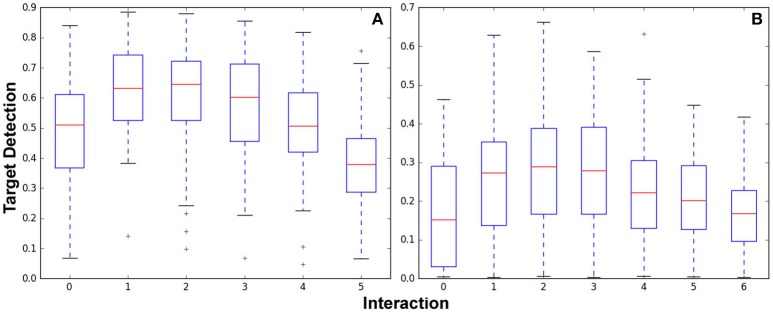
Target detection performance for the Optimizing Interaction as Part of Cost experiments shown in the orthogonal map **(A)** Figure 6 and the complex indoor environment map **(B)** Figure 7.

To examine the effect of the constraints in the “ticking time-bomb” scenario empirically, A large-scale experiment with over 1,200 runs was conducted, and results are shown in Figure 8. For the Figure 6 map, 100 ground-truth priors, 4 elevated areas of target likelihood were generated per prior, were generated and used in simulated interaction experiments. Using a budget *B* = 90, 529 total replanning steps occurred, from 0 to 5 interactions. For the Figure 7 map, 97 ground-truth priors were generated and used in simulated interaction experiments. Because the map in Figure 7 is larger, 7 target likelihood locations were generated per prior. With a budget *B* = 140, 674 total replanning steps occurred, from 0 to 6 interactions.

Figure 8A shows box plots of results from the 529 experiments run after 0–5 interactions. Experiments per interaction were (100, 100, 100, 96, 81, and 52) for (0, 1, 2, 3, 4, and 5) interactions. Figure 8B shows box plots of results from the 674 experiments run after 0-6 interactions. Experiments per interaction were (97, 97, 97, 97, 97, 95, and 94) for 0–6 interactions.

Both graphs show that while interaction initially increases performance as initially demonstrated in section 4, the incorporation of the cost of interaction and replanning results in diminishing returns. In Figure 8A, performance peaks in the one to two interaction range; in Figure 8B, performance is best for two to three interactions. Clearly, the difference in budget as well as size and configuration of the environment has impacts on the number of interactions before the interaction cost begins to impact performance. By incorporating interaction and replanning cost to examine the end-to-end system performance these results show that there is indeed a trade off when leveraging human interaction. This effect should be considered in the creation of any IS3 system for application in a real-world “ticking time-bomb”-type scenario.

## 6. Constraint maintenance in field robotics application

Finally, we demonstrate the IS3 system's ability to perform on a real robot, within constraints per our formulation, by generating tours that stay under budget when executed by a real robot in a field environment.

To improve the adaptability of our system to new partial indicators from interactions and ensure performance under constraints, we wish reserve some unexpended budget in situations where a lower reward does not justify a higher cost expenditure, i.e., “it's not worth the trip.” In real robotics implementations, this will also help account for the potential variability in the cost (time or distance) of the execution of a path relative to the expected cost of the path. To achieve this, we use the same Modified RO approach in section 4.1, with the following modifications to the problem formulation.

### 6.1. Approach

Equation (4) is modified to balance the budget expended to obtain the reward by simultaneously maximizing reward and minimizing cost in the objective function. We rewrite Equation (4) as:

(7) $$\begin{array}{l} \underset{{}_{\text{v}\subset V}}{\arg\max}\;R(v)-\lambda C(v)\\ \text{subject to }C()\leq B\\ \;v=\left[\begin{array}{c} v_{s},\ldots \end{array}\right]. \end{array}$$

where λ is a trade-off parameter controling the cost effect.

The formulation in Equation (4) is particularly useful in iterative, online applications. When searching via Algorithm 2, the cost *C* would likely approach budget *B*, meaning the budget would likely be expended. In these cases, generation of a new, different solution **v**^*k*^ would rely upon delete events (line 10). Because delete events' frequency is proportional to the size of the current solution |v^*^| relative to the entire viewpoint space |*V*|, in cases of increasing environment size, such as real-world field environments, the cost of producing new solutions would also increase. The trade-off parameter λ allows us to balance the likelihood that an initial solution **v**^0^ will have unexpended budget with the information-based reward obtainable. This ensures that future solutions will have increased variability in execution.

### 6.2. Experiments

Here we cover the experiments performed to show performance under constraints in field environments originally presented in Reardon and Fink (2017).

#### 6.2.1. Setup

**Figure 9:**
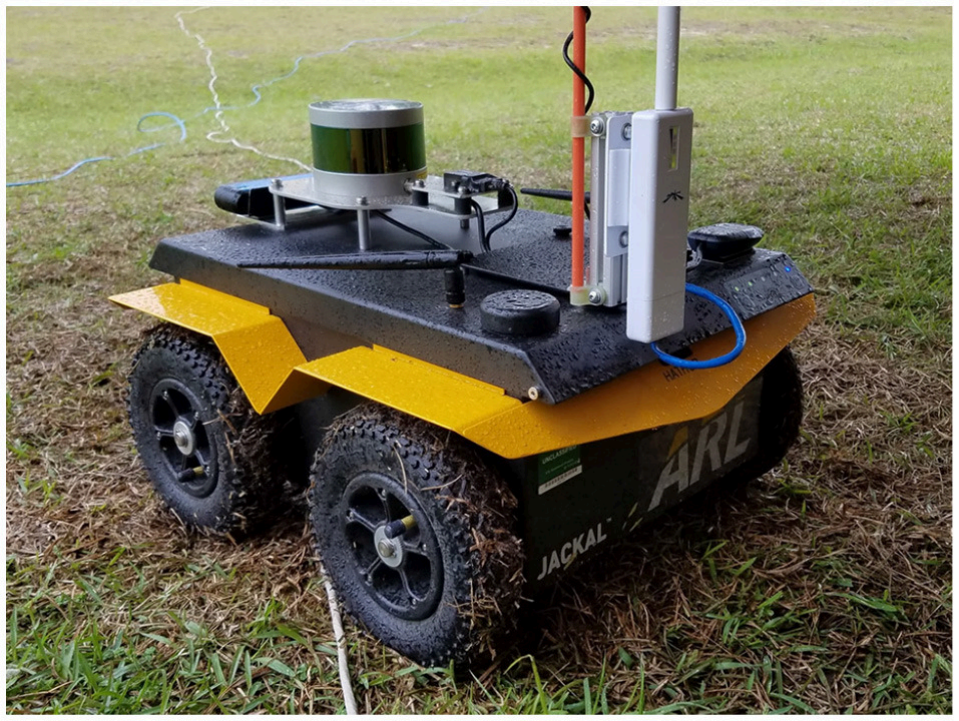
Clearpath Robotics Jackal robot used in the Constraint Maintenance in Field Robotics Application experiments in section 6.2.

The robot used in these experiments is a Clearpath Robotics Jackal (Figure 9) which is a wheeled platform that is limited to a maximum velocity of 1 m/s and is suitable for outdoor operations. It is equipped with a Velodyne VLP-16 LiDAR, which generates a 360° point cloud of 300,000 points per second at a range of 100m and an accuracy of up to ±3 cm. an ASUS Xtion camera for RGB data, a MicroStrain 3DM-GX3-25 IMU and a Garmin 18x PC GPS.

**Figure 10:**
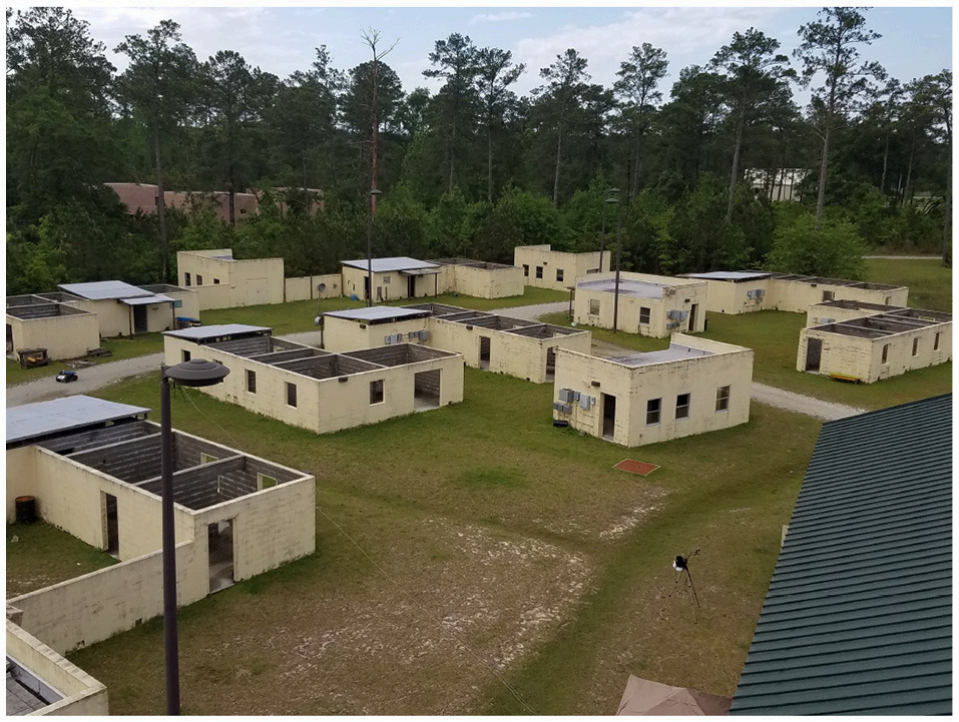
Marketplace environment for the Constraint Maintenance in Field Robotics Application experiments in section 6.2.

We employ onboard custom ROS components for SLAM and optimization-based trajectory control to autonomously navigate through a complex environment as described by Gregory et al. (2016). Our SLAM system, which is specifically designed for operation in GPS denied environments such as building interiors and caves, is a pose-graph framework that uses 3D laser scanners and ICP to build maps of these types of environments and estimate robot trajectory (Rogers et al., 2014). The environment for these experiments consists of multiple concrete buildings and a street arranged and staged as a cluttered village marketplace (Figure 10).

Ten AprilTags (Olson, 2011) were placed throughout the environment to represent targets and AprilTag detection was run on the video stream from the robot. Interactions were performed by a researcher for control; in future work we will explore the broader interaction space with user studies. 12 tours each for baseline (0 interactions), 1, and 2 interactions, were conducted for 36 surveillance tours total. One set was aborted during the *n* = 1 interactions run when the robot failed leaving 35 completed tours in our results set.

Tours were confined to within a 20m radius of the marketplace center. A distance budget *B* = 150*m* and λ = 35 was used. A route was generated after each interaction step (initially no interaction), and the robot used a kinematically feasible motion planner to navigate autonomously. Tour generation took approximately 5–15 s on a computer with an Intel Core i7 2.90GHz Quad Core mobile processor.

#### 6.2.2. Results

**Figure 11:**
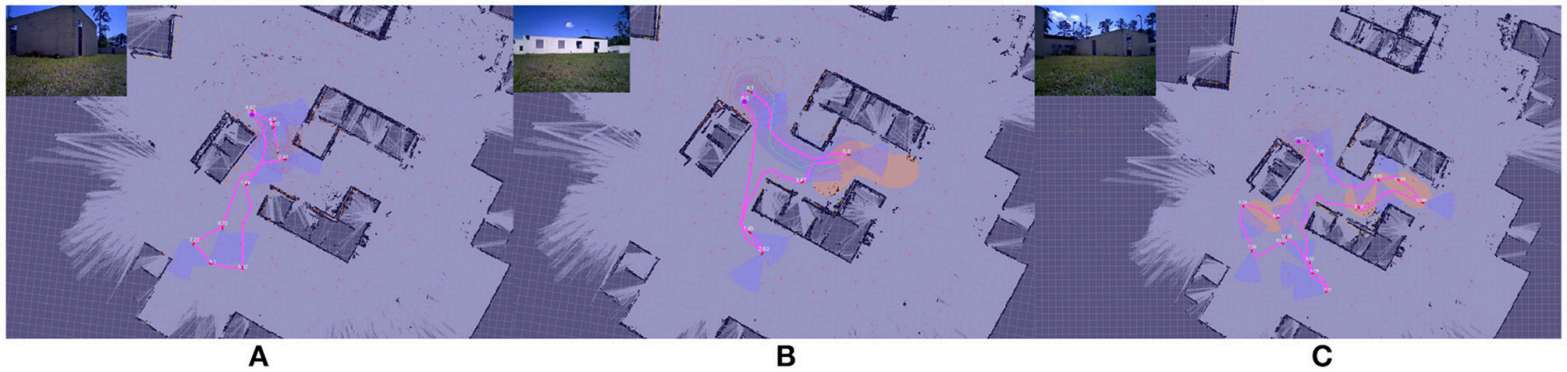
Example illustrative results showing surveillance tour shaping on a real robot in the Constraint Maintenance in Field Robotics Application experiments in section 6.2. Results are visualized in RViz: occupancy grid background is overlayed with generated paths; top left is a video overlay of live robot camera view. **(A)** shows a surveillance tour generated with zero interactions. **(B,C)** show one and two interactions (illustrated by the red areas). Inset images are live video streams on which AprilTag target detection is performed.

An illustrative example of the shaping of the surveillance tour on a real robot is provided in Figure 11, showing zero, one, and two interactions. As can be seen, the path in Figure 11A is shorter than in Figures 11B,C, in which the planner plans tours of increasing length as the opportunity for reward increases. This is a result of the λ trade-off parameter on cost-effect.

**Figure 12:**
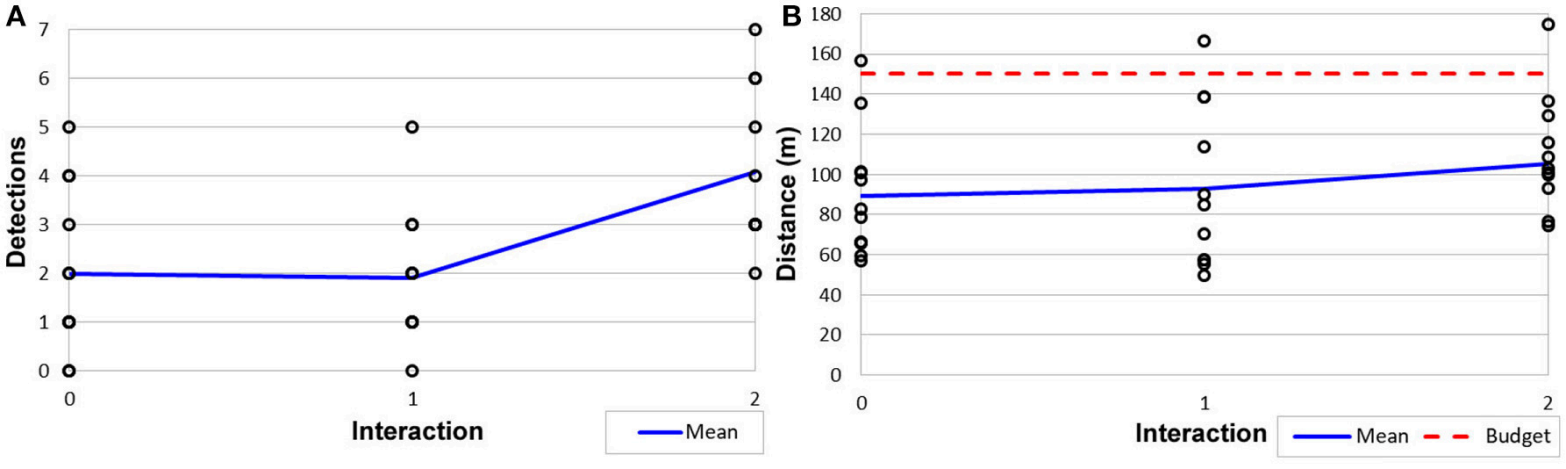
Results from Constraint Maintenance in Field Robotics Application experiments (Section 6.2) showing **(A)** target detections out of 10 possible targets and **(B)** distance traveled and budget.

To determine the ability to perform within constraints, physical distance traveled and unique target detections were calculated for each tour; results are shown in Figure 12. As a result of both the stochastic nature of our route planning algorithm (Algorithm 2) and kinematic plan execution in a real world environment, there is a high degree of variability in the executed path length. Despite this, our trade-off parameter λ ensured that 94% of the planned paths when executed stayed within the budget *B* (Figure 12B), Variability in the orientations achieved by the kinematic motion planner added noise to the target detection; however, we still achieved a statistically significant (*p* < 0.05) increase in the mean number of target detections between 1 and 2 interactions (Figure 12A). We believe both of these real-world issues can be addressed in future work.

## 7. Conclusion

To address the fundamental problem in shared autonomy of efficient problem specification from a human to an autonomous system, we have presented a paradigm, Interactive Shared Solution Shaping (IS3). To examine the general hypothesis that the interaction process can be optimized so that with minimal interactions we can achieve near-optimal results, we have presented results from three different formulations of the IS3 paradigm to three scenarios within the autonomous route planning problem domain.

First, we have shown that the IS3 approach applied to autonomous route planning is able to improve target detection performance and decrease variance. Second, we then build upon this fundamental finding by introducing a formulation to address real-world scenarios where the total end-to-end cost of the system's execution, including interaction, must be accounted for. By incorporating interaction and replanning time we identify the existence of an optimal number of interactions vs. performance in an end-to-end system. We believe future work could seek to both derive and optimize over this utility function. Finally, we show how our system applies to a real robotic application. We introduce a trade-off parameter to balance cost against reward and better maintain constraints, and implement our approach on a robot in a real-world setting and show that our approach not only works but is able to perform within budget constraints.

Collectively, the work presented here exhibits the concept of interactive shaping of a solution space between a human and an autonomous system. We believe the scenarios illustrate specific instances of a broader concept that could apply to many other complex application domains where a human has information that could improve the performance of an autonomous agent but which cannot be fully specified. We plan to examine this concept further with large-scale user studies in future work.

## Author contributions

All authors contributed to the theoretical approach presented. In addition, JF and CR contributed to the experimental validation.

### Conflict of interest statement

The authors declare that the research was conducted in the absence of any commercial or financial relationships that could be construed as a potential conflict of interest.
